# The burden and risk factors of chronic obstructive pulmonary disease in Asia and its countries from 1990 to 2021: a systematic analysis based on the 2021 global burden of disease study

**DOI:** 10.3389/fmed.2025.1641719

**Published:** 2025-09-19

**Authors:** Zeyuan Wu, Xin Zhang, Pingxi Zhang, Yaling He, Yalin Ye, Yun Pan, Yan Li

**Affiliations:** ^1^School of Basic Medicine, Dali University, Dali, China; ^2^Department of Pathology, The First Affiliated Hospital of Dali University, Dali, China

**Keywords:** chronic obstructive pulmonary disease (COPD), disease burden, risk factors, Socio-demographic Index (SDI), Joinpoint regression

## Abstract

**Objective:**

Chronic obstructive pulmonary disease (COPD) is a chronic respiratory disease characterized by airflow limitation that is not fully reversible. In Asia, risk exposures such as smoking and ambient PM₂.₅ are prevalent, and regional differences are significant. This has led to the COPD disease burden in this region being at a relatively high level globally. Based on this, this study conducts a systematic assessment of the COPD disease burden in Asia.

**Methods:**

Data on incidence, mortality, and disability-adjusted life years (DALYs) were obtained from the Global Burden of Disease (GBD) 2021 study. The analysis incorporated the Sociodemographic Index (SDI) and included stratification by sex and age to reveal the distribution and disparities in the burden of COPD across populations. Joinpoint regression models were used to calculate the annual percent change (APC) and average annual percent change (AAPC) to estimate temporal trends. In addition, major attributable risk factors for COPD were analyzed to identify key drivers of disease burden across regions and populations.

**Results:**

In 2021, the number of COPD cases in Asia reached 10,512,843 (95% UI: 9,610,006–11,432,970), with an age-standardized incidence rate of 210.79 per 100,000 persons (95% UI: 193.52–227.94). COPD accounted for 2,885,059 deaths (95% UI: 2,571,267–3,218,689), corresponding to an age-standardized mortality rate of 64.10 per 100,000 persons (95% UI: 56.74–71.66). The total DALYs were 60,507,100 (95% UI: 55,319,463–66,518,282), with an age-standardized rate of 1,253.15 per 100,000 persons (95% UI: 1,148.26–1,376.29). Among Asian subregions, South Asia bore the heaviest burden of COPD. Major risk factors included particulate matter (PM) pollution, smoking, secondhand smoke, and occupational exposure to particulate matter, gases, and fumes (OP-MGF).

**Conclusion:**

This study highlights the substantial COPD burden in Asia, with air pollution, smoking, and occupational exposures being the predominant risk factors. Targeted public health interventions are urgently needed to mitigate the COPD burden and improve overall health outcomes in the region.

## 1 Introduction

Chronic obstructive pulmonary disease (COPD) is one of the most prevalent chronic respiratory diseases worldwide. It has long ranked among the leading causes of death from chronic conditions, posing a heavy burden on both public health and socioeconomic systems (1, 2). In Asia, due to the combined effects of demographic structure and environmental exposures, the prevalence and mortality of COPD are significantly higher than the global average (3). For developing countries with limited health resources, this disparity underscores the importance of formulating region-specific prevention and control strategies (4, 5).

Patients with COPD commonly present with chronic cough, sputum production, and exertional dyspnea. Recurrent acute exacerbations accelerate disease progression and lead to persistent decline in lung function, thereby reducing quality of life and increasing healthcare burden (5). The major risk factors for COPD include smoking, outdoor air pollution, indoor solid fuel use, and occupational exposures, all of which are closely related to lifestyle and socioeconomic status (6). In Asia, widespread biomass fuel use in rural areas of South and Southeast Asia poses particular harm to women and children, while rapid urbanization and industrialization have led to sustained high levels of PM₂.₅ exposure and increased risks of occupational dust (7). The combination of these factors has substantially exacerbated the regional disease burden, highlighting the necessity of targeted research and intervention.

Although previous studies have systematically evaluated the long-term global trends and risk factors of COPD, research focusing on regional and inter-country differences in disease burden within Asia remains limited. Based on data from the Global Burden of Disease (GBD) 2021 study, this research assessed age-standardized rates (ASRs) and their temporal trends for COPD across Asian regions, age groups, sexes, and countries from 1990 to 2021. The analysis was further stratified by the Sociodemographic Index (SDI) to reveal disparities in disease burden across different levels of socioeconomic development. In addition, Joinpoint regression models were applied to calculate the annual percent change (APC) and average annual percent change (AAPC) for various periods. This study aims to systematically characterize the distribution and temporal evolution of the COPD burden in Asia, providing scientific evidence to guide public health interventions.

## 2 Methods

### 2.1 Data source

The Global Burden of Disease (GBD) database,^1^ developed through the collaboration of the Institute for Health Metrics and Evaluation and its global partners, is a comprehensive health data platform designed to evaluate the impact of diseases, injuries, and associated risk factors on health at the global, regional, and national levels (8, 9). This database covers multiple dimensions of health metrics, including mortality, incidence, disability-adjusted life years (DALYs), and risk factors (10). Data from the GBD 2021 study were retrieved through the VizHub–GBD Results tool.^2^ The classification of Asian countries and regions followed the definitions used in the GBD 2021 database, and the complete list of Asian countries included in this study is provided in Supplementary Table S1.

### 2.2 Trend analysis: Joinpoint regression model

We applied the Joinpoint regression model to fit the time-series data. The primary objective of this model is to identify statistically significant time points at which the trend changes, referred to as “joinpoints” or “turning points,” and to quantify the rate of change within each time segment (11). Based on this framework, we calculated the annual percent change (APC) and the average annual percent change (AAPC). A positive trend was considered statistically significant when the lower limit of the 95% confidence interval (CI) of APC or AAPC was greater than 0, whereas a negative trend was considered statistically significant when the upper limit of the 95% CI was less than 0 (6). The specific calculation formula is as follows:


$$\mathrm{APC}=(e^{\beta }-1)\times 100\%,\text{AAPC}=(e^{\sum \mathrm{w\beta}}-1)\times 100\%$$

In this formula, *β* represents the regression slope for each time segment, and *w* denotes the weight, reflecting the proportion of that segment within the entire study period (12).

### 2.3 Socio-demographic Index

In this study, countries at different levels of the Socio-demographic Index (SDI) were selected for specific analyses. The SDI is a composite indicator that reflects the level of socioeconomic development in a region, primarily incorporating income, human capital (such as educational attainment), and life expectancy (13, 14). By comparing the COPD burden across regions with varying SDI levels, this study aimed to elucidate the influence of socioeconomic development on COPD burden.

### 2.4 Standardized assessment of disease burden

We evaluated the COPD burden using age-standardized rates (ASRs), including the age-standardized incidence rate (ASIR), age-standardized mortality rate (ASMR), and age-standardized disability-adjusted life year rate (AS-DALY). The ASR was calculated using the following formula:


$$\mathrm{ASR}=\frac{\sum_{i=1}^{k}\;w_{i}r_{i}}{\sum_{i=1}^{k}\;w_{i}}$$

In this formula, i represents the age group, w denotes the population size of the standard population in the corresponding age group, and k refers to the total number of age groups. According to the GBD framework, all point estimates were obtained by averaging 1,000 draws, while the 95% uncertainty interval (UI) was defined as the 2.5th and 97.5th percentiles of the distribution of these draws, thereby providing a more comprehensive reflection of the uncertainty in the estimates (15).

### 2.5 Statistical analysis

Joinpoint regression analyses were performed using the Joinpoint Regression Program (version 5.30). Data preprocessing and visualization were conducted in R (version 4.3.2). These tools ensured the accuracy and reproducibility of the analyses. For outcomes derived from the GBD database (ASIR, ASMR, and AS-DALY), we reported 95% uncertainty intervals (95% UIs). For trend indicators obtained from Joinpoint regression (APC and AAPC), we reported 95% confidence intervals (95% CIs) and *p*-values. These two types of intervals reflect different sources of statistical uncertainty, corresponding to sampling uncertainty in the GBD framework and inferential uncertainty from regression modeling, respectively.

## 3 Results

### 3.1 Global burden analysis from 1990 to 2021

**Figure 1:**
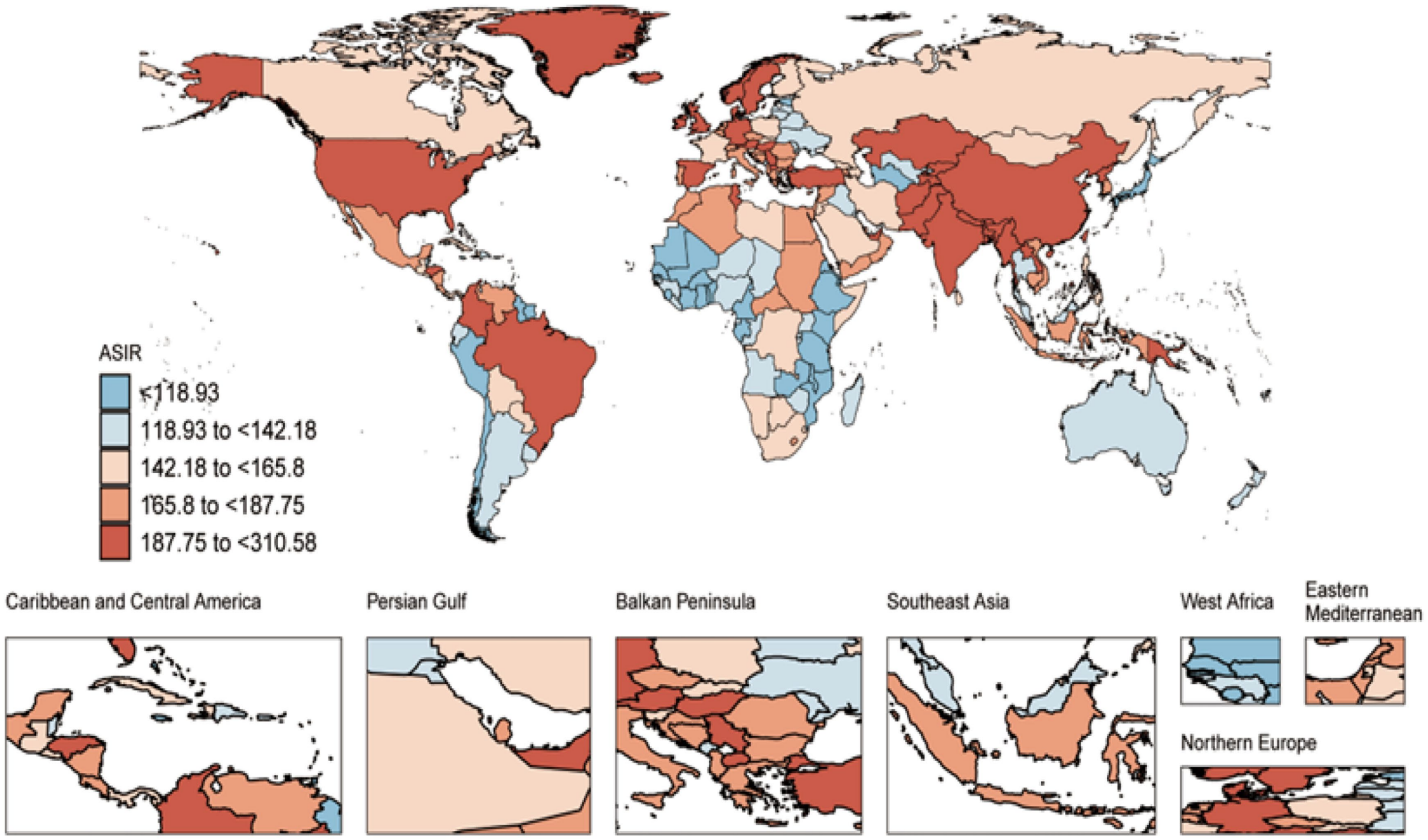
Global distribution of COPD burden in 2021: age-standardized incidence rate (ASIR).

In 2021, the global incidence of COPD was 16,895,445 cases (95% UI: 15,471,347–18,335,691), with an age-standardized incidence rate (ASIR) of 197.37 per 100,000 persons (95% UI: 181.65–213.42). From 1990 to 2021, the ASIR decreased by 0.02 per 100,000 persons (95% UI: −0.05 to −0.01) (Figure 1 and Supplementary Table S2). The number of COPD-related deaths was 3,719,937 (95% UI: 3,347,912–4,084,218), corresponding to an age-standardized mortality rate (ASMR) of 45.22 per 100,000 persons (95% UI: 40.61–49.70). Between 1990 and 2021, the ASMR declined by 0.37 per 100,000 persons (95% UI: −0.43 to −0.28) (Figure 1 and Supplementary Table S2). The total number of disability-adjusted life years (DALYs) was 79,779,695 (95% UI: 74,026,373–86,011,406), with an age-standardized DALY rate (AS-DALY) of 940.66 per 100,000 persons (95% UI: 871.48–1,014.59). From 1990 to 2021, the AS-DALY rate decreased by 0.37 per 100,000 persons (95% UI: −0.42 to −0.29) (Figure 1 and Supplementary Table S2).

### 3.2 Correlation analysis with SDI

In 2021, across 48 Asian countries with different SDI levels, COPD showed significant negative correlations with the ASIR, ASMR, and AS-DALY (ASIR: R = −0.53, *p* < 0.001; ASMR: R = −0.66, *p* < 0.001; AS-DALY: R = −0.69, *p* < 0.001) (Supplementary Figure S2). Here, *R* denotes the correlation coefficient, and *p* < 0.05 was considered statistically significant.

**Figure 2:**
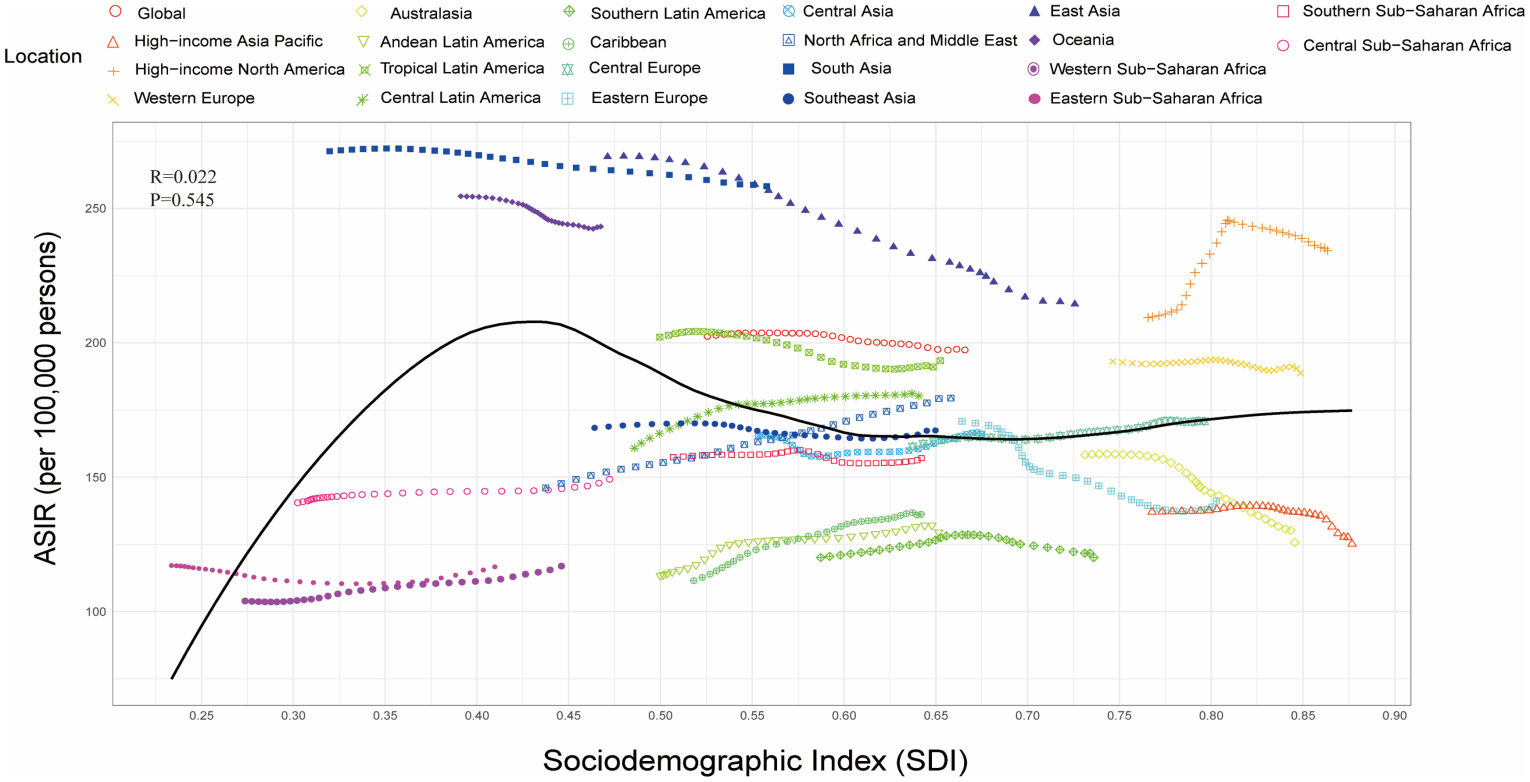
Relationship between the Socio-demographic Index (SDI) and the age-standardized incidence rate (ASIR) of chronic obstructive pulmonary disease (COPD) across 22 regions, 1990–2021.

Analysis of COPD burden across 22 global regions stratified by SDI from 1990 to 2021 indicated a positive but non-significant correlation between ASIR and SDI (ASIR: R = 0.022, *p* = 0.545) (Figure 2), whereas ASMR and AS-DALY were significantly negatively correlated with SDI (Supplementary Figure S3). Specifically, in Asia, both East Asia and South Asia exhibited ASMR and AS-DALY values higher than expected relative to their SDI levels. Even at relatively high SDI levels, the COPD burden in East and South Asia remained substantially greater than predicted (Supplementary Figure S3), underscoring the severity of the COPD burden in these regions.

### 3.3 Risk factor analysis

**Figure 3:**
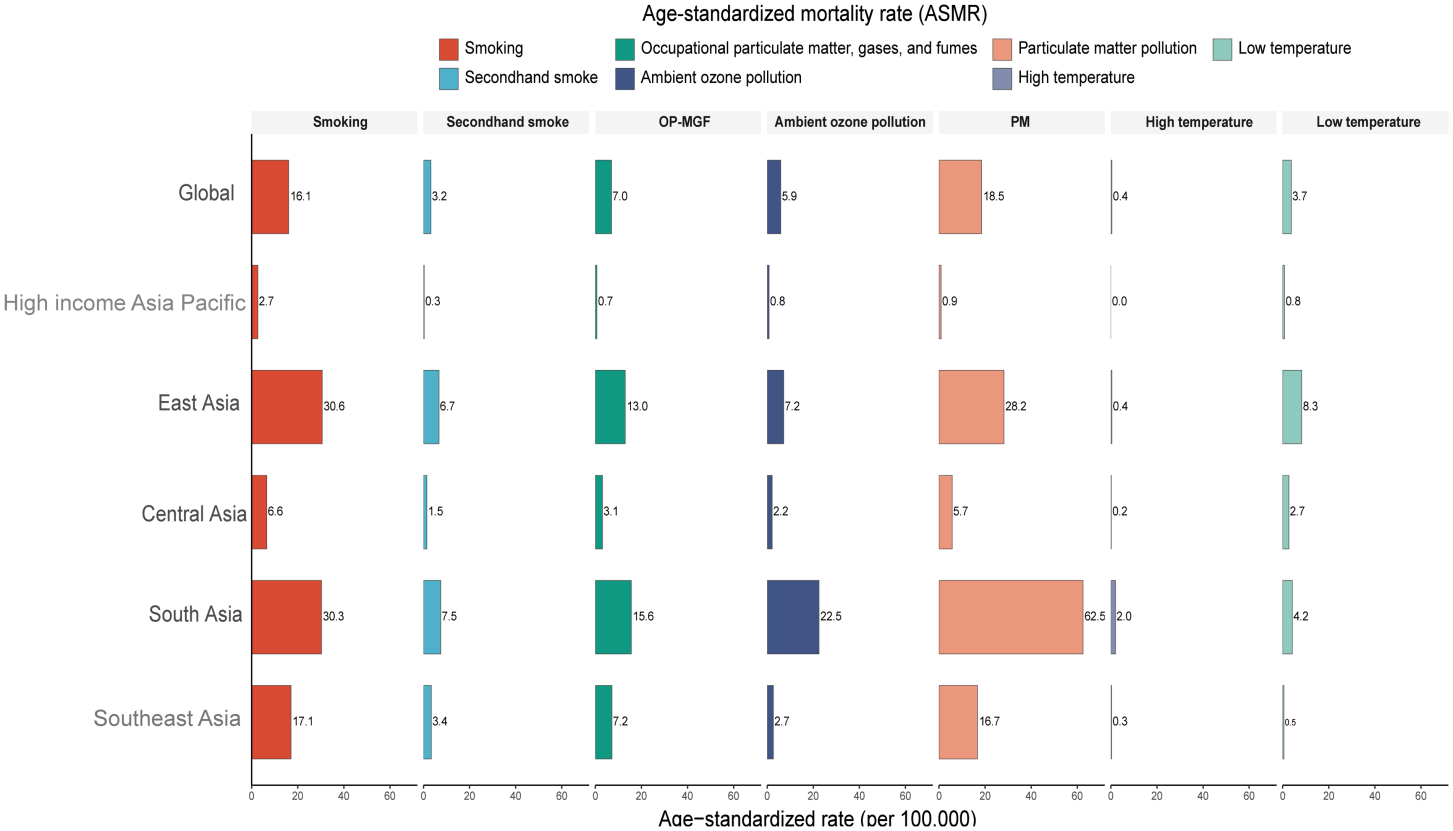
Proportion of age-standardized mortality rate (ASMR) attributable to risk factors for chronic obstructive pulmonary disease (COPD) in Asia in 2021. Risk factors include smoking, secondhand smoke, occupational exposure to particulate matter, gases, and fumes (OP-MGF), ambient ozone pollution, particulate matter (PM) pollution, high temperature, and low temperature.

In South Asia, COPD mortality rates attributable to ambient ozone pollution, high temperature, and occupational particulate matter, gases, and fumes (OP-MGF) were the highest among all Asian regions (Figure 3). In contrast, in East Asia, COPD mortality related to smoking and low temperature was the most severe in the region (Figure 3).

The impact of different risk factors on COPD-related DALYs in Asia also warrants attention. In 2021, South Asia had the highest COPD DALY burden attributable to smoking, OP-MGF, high temperature, and ambient ozone pollution (Supplementary Figure S4). East Asia, however, showed the highest DALY burden associated with low temperature (Supplementary Figure S4).

**Figure 4:**
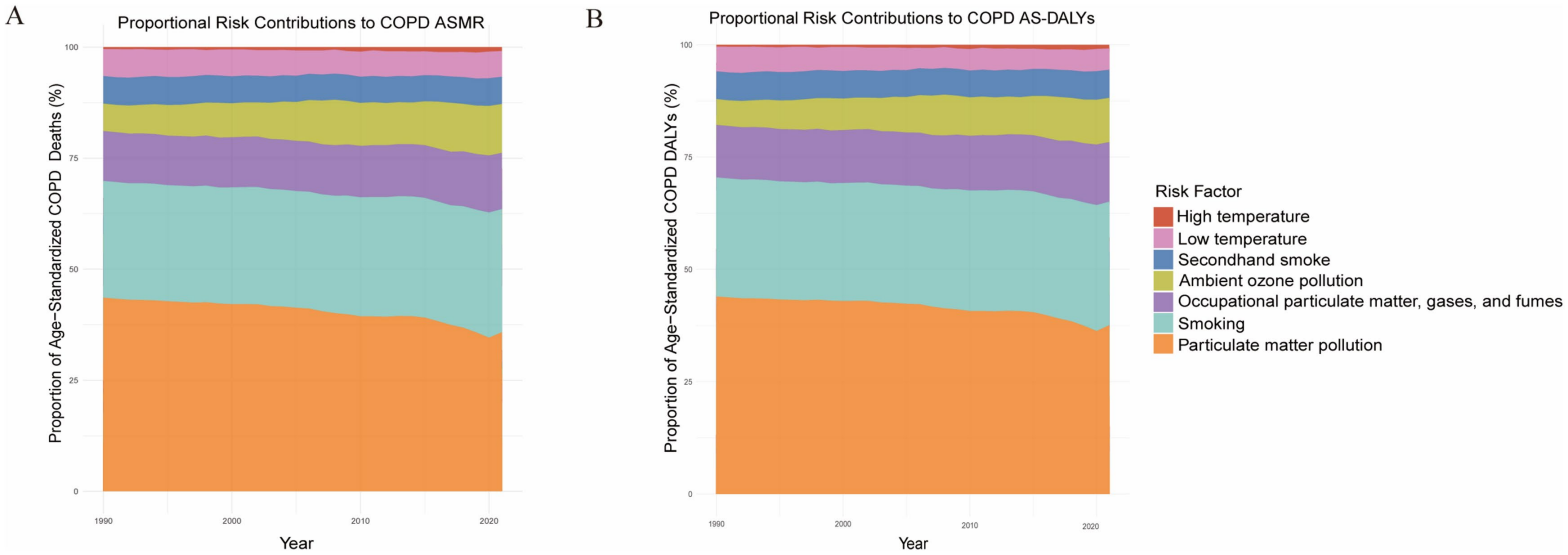
Proportional contributions of major risk factors to the age-standardized mortality rate (ASMR) and age-standardized disability-adjusted life year rate (AS-DALY) for chronic obstructive pulmonary disease (COPD) in Asia, 1990–2021. **(A)** Proportional contributions of major risk factors to COPD ASMR. **(B)** Proportional contributions of major risk factors to COPD AS-DALY.

Finally, we analyzed the age-standardized attributable fractions of COPD mortality and DALY rates due to various risk factors in Asia. The results indicated that particulate matter pollution, smoking, and secondhand smoke were the predominant contributors to COPD burden, accounting for the largest proportions, which suggests their critical role in the development and progression of COPD (Figures 4A,B). In comparison, the contributions of occupational exposures, ozone pollution, and extreme temperatures (both high and low) were relatively smaller and remained largely stable from 1990 to 2020 (Figures 4A,B). Nevertheless, despite their smaller contributions overall, these factors may still exert significant localized effects in specific populations or regions.

### 3.4 Asia level

**Figure 5:**
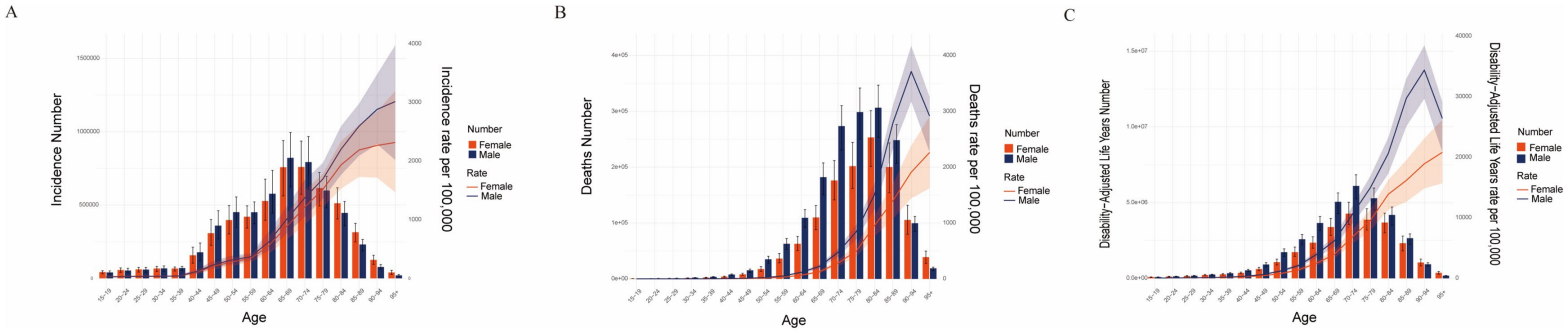
Sex and age-structured analysis of COPD burden in Asia in 2021. **(A)** Age-standardized incidence rate (ASIR) and case numbers. **(B)** Age-standardized mortality rate (ASMR) and death numbers. **(C)** Age-standardized disability-adjusted life year rate (AS-DALY) and DALY numbers.

In 2021, age- and sex-specific analyses revealed significant differences in the COPD burden across Asia. The incidence began to increase after the age of 40, while mortality rose markedly after the age of 55. Overall, men consistently exhibited a higher disease burden than women (Figures 5A–C).

In 2021, South Asia recorded the highest ASIR, ASMR, and AS-DALY among all Asian regions (Table 1). From 1990 to 2021, East Asia demonstrated the greatest reductions in ASIR, ASMR, and AS-DALYs across Asia (Table 1).

**Table 1:** Incident cases, death cases, and DALYs of COPD in 2021 by sex, and rate changes of age-standardized rates across Asian regions (Global Burden of Disease, GBD).

Region	Incidence (95% uncertainty interval)			Deaths (95% uncertainty interval)			DALYs (95% uncertainty interval)		
	Counts (2021)	Age-standardised rates per 100,000 (2021)	Rate change in age-standardised rates, 1990–2021	Counts (2021)	Age-standardised rates per 100,000 (2021)	Rate change in age-standardised rates, 1990–2021	Counts (2021)	Age-standardised rates per 100,000 (2021)	Rate change in age-standardised rates, 1990–2021
Asia									
Both	1,05,12,843	210.79	−0.10	28,85,059	64.10	−0.50	6,05,07,100	1253.15	−0.48
	(9,610,006–11,432,970)	(193.52–227.94)	(−0.13 to −0.07)	(2,571,267–3,218,689)	(56.74–71.66)	(−0.56 to −0.40)	(55,319,463–66,518,282)	(1148.26–1376.29)	(−0.54 to −0.39)
Male	52,88,933	223.78	−0.09	16,65,024	84.76	−0.45	3,46,78,170	1562.09	−0.45
	(4,839,008–5,744,380)	(206.88–241.03)	(−0.12 to −0.06)	(1,402,706–1,862,251)	(71.08–94.66)	(−0.55 to −0.35)	(29,894,694–38,510,138)	(1350.26–1731.93)	(−0.55 to −0.36)
Females	52,23,910	199.98	−0.11	12,20,034	48.63	−0.54	2,58,28,930	999.58	−0.51
	(4,742,523–5,689,254)	(182.19–217.15)	(−0.14 to −0.08)	(995,462–1,468,103)	(39.58–58.56)	(−0.64 to −0.37)	(21,901,316–30,064,248)	(848.33–1201.24)	(−0.59 to −0.35)
East Asia									
Both	45,67,271	214.45	−0.20	13,23,441	72.20	−0.68	2,43,91,744	1217.69	−0.68
	(4,133,512–5,000,602)	(197.24–233.32)	(−0.25 to −0.17)	(1,082,508–1,574,211)	(59.32–85.26)	(−0.74 to −0.60)	(20,763,410–28,628,224)	(1043.87–1422.79)	(−0.74 to −0.60)
Male	22,91,903	228.17	−0.16	7,70,633	103.24	−0.63	1,38,64,925	1581.54	−0.64
	(2,085,274–2,504,020)	(210.59–245.58)	(−0.21 to −0.13)	(605,702–935,956)	(82.62–123.32)	(−0.73 to −0.53)	(11,033,302–16,728,478)	(1272.87–1887.92)	(−0.74 to −0.55)
Females	22,75,368	203.16	−0.24	5,52,808	52.28	−0.73	1,05,26,819	958.49	−0.71
	(2,051,160–2,504,686)	(184.61–222.81)	(−0.29 to −0.20)	(413,884–708,340)	(39.00–66.78)	(−0.81 to −0.60)	(8,388,404–13,073,388)	(766.98–1187.94)	(−0.78 to −0.58)
South Asia									
Both	37,79,866	258.26	−0.05	12,29,609	101.63	−0.11	2,80,09,599	2049.22	−0.16
	(3,513,161–4,030,885)	(242.49–273.77)	(−0.07 to −0.03)	(1,104,445–1,381,284)	(90.55–114.34)	(−0.25 to 0.15)	(25,404,007–30,942,201)	(1862.71–2268.73)	(−0.27 to 0.06)
Male	18,13,052	254.73	−0.06	6,80,330	117.57	−0.12	1,56,46,367	2351.16	−0.16
	(1,684,998–1,940,439)	(238.62–269.97)	(−0.08 to −0.03)	(551,479–782,547)	(96.18–135.33)	(−0.27 to 0.13)	(12,777,218–17,747,814)	(1929.06–2665.91)	(−0.29 to 0.06)
Females	19,66,814	262.11	−0.04	5,49,279	87.30	−0.06	1,23,63,233	1766.17	−0.11
	(1,828,839–2,093,183)	(245.44–277.67)	(−0.06 to −0.02)	(430,865–669,885)	(68.60–106.52)	(−0.28 to 0.66)	(10,084,776–14,609,562)	(1438.88–2092.84)	(−0.29 to 0.41)
Central Asia									
Both	1,33,302	166.10	0.003	14,472	21.27	−0.41	3,85,341	498.60	−0.39
	(118,936–148,131)	(148.28–184.13)	(−0.04 to 0.06)	(12,972–16,004)	(19.12–23.46)	(−0.47 to −0.35)	(348,373–424,298)	(685.79–776.50)	(−0.44 to −0.32)
Male	59,459	172.02	−0.04	8,724	32.04	−0.43	2,27,352	689.67	−0.42
	(53,112–66,056)	(154.70–190.11)	(−0.09 to 0.01)	(7,828–9,700)	(28.98–35.34)	(−0.49 to −0.36)	(203,951–252,666)	(623.67–762.50)	(−0.48 to −0.35)
Females	73,843	163.25	0.03	5,748	14.39	−0.44	1,57,989	362.49	−0.38
	(66,105–82,315)	(146.05–181.95)	(−0.03 to 0.10)	(5,079–6,574)	(12.74–16.41)	(−0.50 to −0.36)	(141,748–178,396)	(325.41–406.23)	(−0.44 to −0.30)
Southeast Asia									
Both	10,70,750	167.41	−0.01	2,28,487	43.14	−0.28	55,58,590	914.73	−0.27
	(953,956–1,190,856)	(149.52–185.92)	(−0.03 to 0.02)	(202,410–257,525)	(38.24–48.54)	(−0.39 to −0.03)	(4,982,450–6,206,668)	(822.27–1016.35)	(−0.37 to −0.05)
Male	5,94,556	201.01	−0.0003	1,49,336	65.11	−0.25	36,31,416	1326.47	−0.24
	(536,762–660,123)	(181.21–221.06)	(−0.03 to 0.03)	(132,444–167,958)	(58.10–72.82)	(−0.37 to −0.001)	(3,237,727–4,063,625)	(1186.54–1480.93)	(−0.35 to −0.03)
Females	4,76,194	139.52	−0.01	79,151	26.96	−0.33	19,27,175	587.49	−0.31
	(417,878–537,737)	(122.66–156.75)	(−0.04 to 0.02)	(65,250–99,626)	(22.26–33.77)	(−0.48 to 0.18)	(1,636,485–2,314,606)	(498.41–705.42)	(−0.46 to 0.10)

At the national level, Nepal reported the highest COPD burden in 2021, with an ASIR of 310.58 per 100,000 persons (95% UI: 300.58–319.70), an ASMR of 146.13 per 100,000 persons (95% UI: 116.66–182.46), and an AS-DALY rate of 2,836.01 per 100,000 persons (95% UI: 2,275.30–3,485.04). North Korea and India ranked next (Supplementary Tables S3–S5). Between 1990 and 2021, Singapore exhibited the most pronounced declines in all three indicators, with the ASIR decreasing by −0.37 per 100,000 persons (95% UI: −0.45 to −0.27), the ASMR declining by −0.85 per 100,000 persons (95% UI: −0.87 to −0.84), and the AS-DALY rate dropping by −0.81 per 100,000 persons (95% UI: −0.83 to −0.79). These findings highlight Singapore’s remarkable achievements in COPD prevention and management (Supplementary Table S6).

### 3.5 Trends from 1990 to 2021

**Figure 6:**
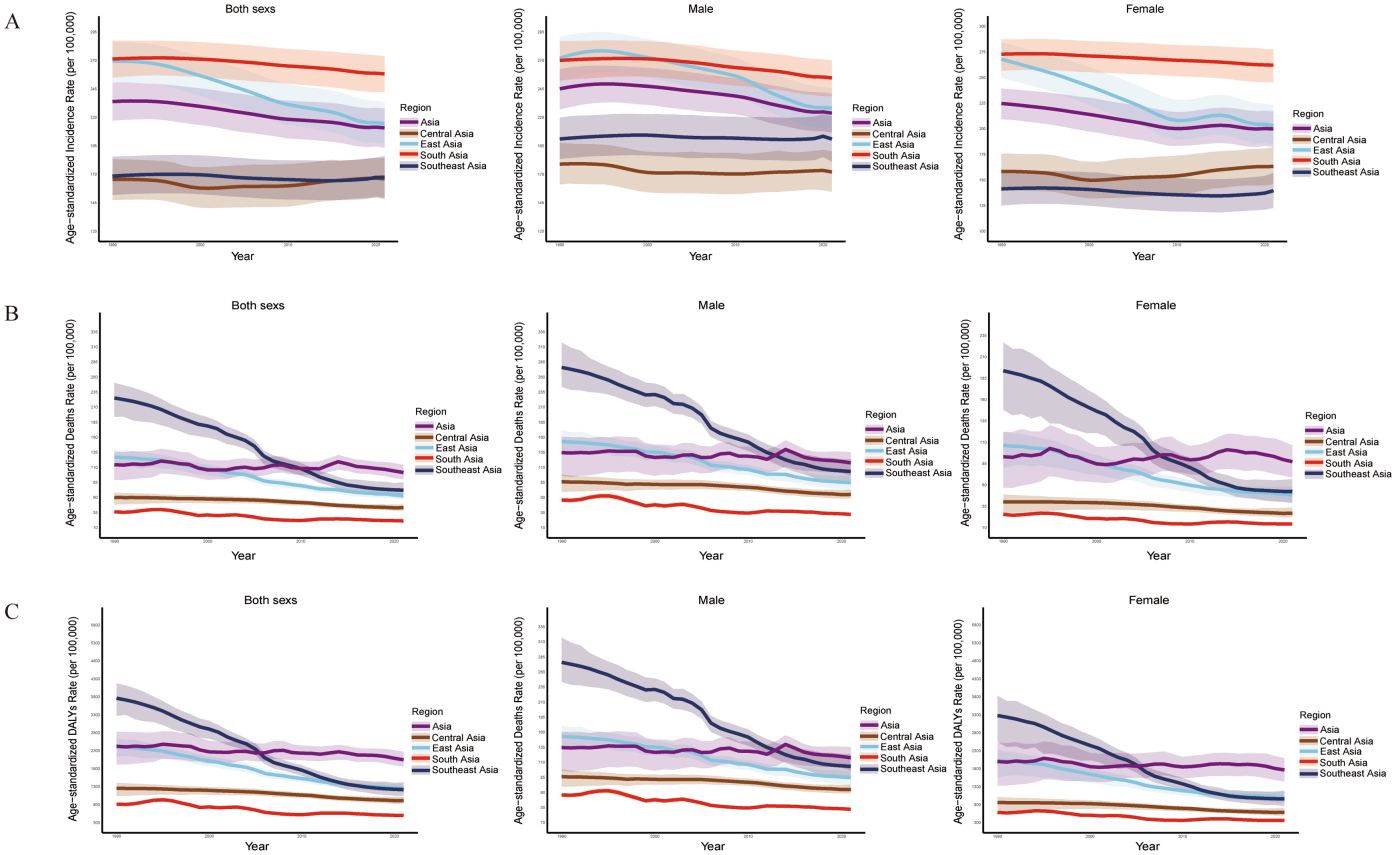
Burden of COPD across sex groups in Asian regions, 1990–2021. **(A)** Age-standardized incidence rate (ASIR). **(B)** Age-standardized mortality rate (ASMR). **(C)** Age-standardized disability-adjusted life year rate (AS-DALY).

From 1990 to 2021, South Asia consistently carried the heaviest COPD burden in Asia, with the highest ASIR observed across all sex groups (male, female, and overall) (Table 1 and Figure 6A). This finding further highlights the severity and complexity of the COPD challenge in this region. In contrast, East Asia demonstrated significant declines in ASMR and AS-DALYs across all sex groups, suggesting remarkable progress in public health policies, treatment, and control measures (Table 1 and Figures 6B,C).

To further explore the influence of sex and age on the COPD burden in Asia, we analyzed the distribution of COPD-related indicators by sex and age group from 1990 to 2021. The analysis included age groups starting from 20 years, stratified at 5-year intervals, and systematically examined incidence, mortality, and DALYs across each group. The results showed that, apart from the “all ages” group, which exhibited increases in incidence, mortality, and DALYs, most other age groups demonstrated varying degrees of decline. Moreover, across all age groups, the disease burden in men remained consistently higher than in women (Supplementary Figures S5–S7).

### 3.6 Joinpoint regression analysis

**Figure 7:**
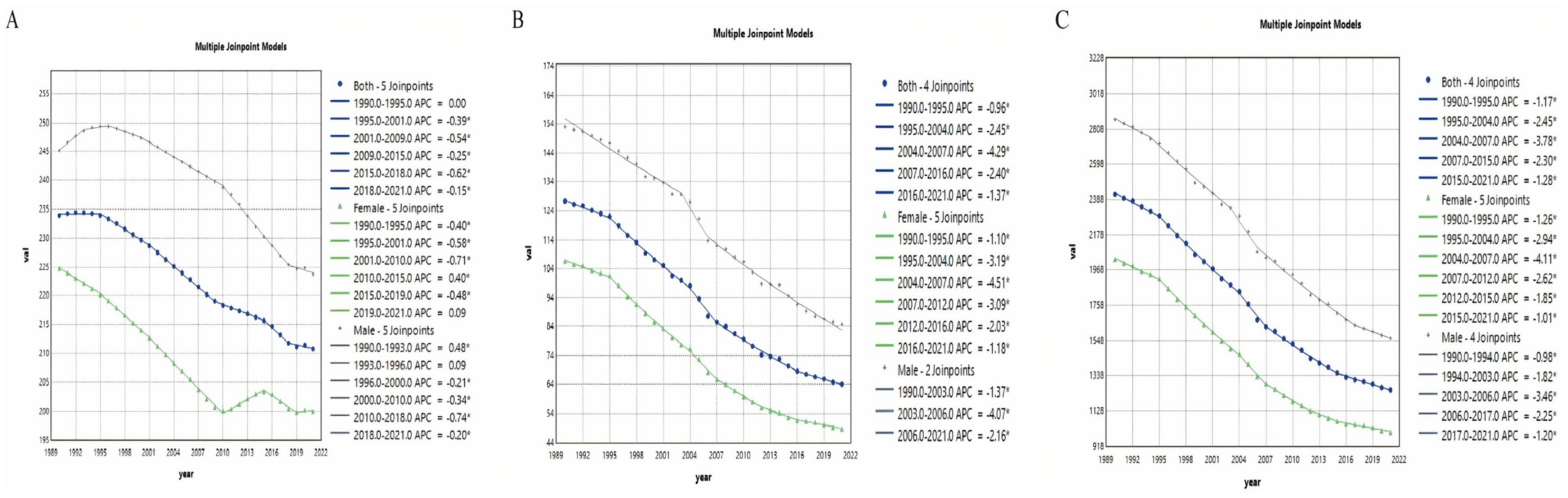
Joinpoint regression analysis of temporal trends in COPD burden, 1990–2021. **(A)** Age-standardized incidence rate (ASIR). **(B)** Age-standardized mortality rate (ASMR). **(C)** Age-standardized disability-adjusted life year rate (AS-DALY).

We conducted Joinpoint regression analyses to examine the temporal trends of COPD burden in Asia. Between 1990 and 2021, the main COPD indicators showed overall downward trends, although the rates of decline and significant periods of change varied. The ASIR demonstrated a decreasing trend (AAPC = −0.338, 95% CI: −0.362 to −0.314, *p* < 0.001) (Supplementary Table S7). The most pronounced decline occurred during 2015–2018 (APC = −0.616, 95% CI: −0.818 to −0.413, *p* < 0.001), indicating a substantial reduction in COPD burden during this period (Figure 7A and Supplementary Table S8).

Both the ASMR and AS-DALY exhibited significant overall decreases (ASMR: AAPC = −2.203, 95% CI: −2.397 to −2.009, *p* < 0.001; AS-DALY: AAPC = −2.112, 95% CI: −2.212 to −2.012, *p* < 0.001) (Supplementary Tables S9, S11). The steepest declines were observed between 2004 and 2007 (ASMR: APC = −4.291, 95% CI: −5.871 to −2.684, *p* < 0.001; AS-DALY: APC = −3.782, 95% CI: −4.611 to −2.946, *p* < 0.001) (Figures 7B,C and Supplementary Tables S10, S12). Overall, the reductions in COPD-related mortality and health loss in Asia were especially notable, although the magnitude of decline varied across different indicators and time periods.

## 4 Discussion

Based on GBD 2021 data, this study systematically analyzed the burden of COPD in Asia. In 2021, the number of new COPD cases exceeded 10 million (16), underscoring that COPD is not only a major chronic respiratory problem but also reflects the profound impact of demographic changes and combined environmental exposures on public health (17). Previous studies have provided a clear picture of the global burden of COPD and the attributable burdens of its major risk factors; however, Asia has often been treated as a single region, with limited characterization of within-region heterogeneity (18). Here, we refine the analytical scale to the country and subregional levels within Asia, identify high-burden hotspots in relation to major risk factors, and—through age- and sex-stratified analyses—delineate priority populations, thereby providing evidence to support region-specific prevention and resource allocation.

The main risk factors for COPD in Asia in 2021 included particulate matter (PM) pollution, smoking, and occupational exposure to particulate matter, gases, and fumes (OP-MGF), with varying contributions across regions. In South Asia, the use of biomass fuels in rural households has caused indoor air pollution that particularly harms women and children, significantly increasing COPD risk (19, 20). In addition, widespread tobacco consumption in India and several other Asian countries has further exacerbated the COPD burden (21). From 1990 to 2021, East Asia experienced a marked reduction in COPD burden. For example, in China, coal control policies, tobacco-control legislation, and the promotion of lung function screening collectively reduced exposures and improved disease management outcomes (22, 23). The East Asian experience demonstrates that comprehensive interventions are critical for COPD prevention and control, providing lessons for other regions (24).

Our 2021 analysis revealed a significant negative correlation between the Sociodemographic Index (SDI) and both ASMR and AS-DALYs in Asia. This trend highlights the importance of public health interventions such as smoking control, air pollution mitigation, and improvements in healthcare services in reducing disease burden (25, 26). However, despite relatively high SDI levels in some countries of East and South Asia, their COPD burden remained higher than expected, suggesting the combined effects of persistently high smoking prevalence, environmental particulate exposure, occupational hazards, and continued use of biomass fuels among certain populations (27). Further analyses showed that from 1990 to 2021, COPD burden in Asian countries was significantly negatively correlated with SDI, yet countries such as Nepal and India exhibited substantially higher burdens than predicted. This discrepancy reflects the critical roles of sociocultural factors, public health policy implementation, and healthcare accessibility in shaping disease burden in these regions (28, 29).

At the national level, the COPD burden in India and Nepal was particularly pronounced. In India, multiple factors, including high smoking prevalence, employment in high-risk industries, and limited public health awareness, have contributed to its leading COPD burden (30). In Nepal, 59.4% of COPD patients were current smokers, and 76.2% relied on traditional firewood for cooking; combined with poor ventilation in cold, high-altitude settings, this exacerbated indoor pollution alongside environmental PM₂.₅ exposure (31). Parallel promotion of tobacco control and clean energy alternatives, together with stronger occupational protections, is urgently required.

In Central Asia, countries such as Kyrgyzstan and Tajikistan—among the poorest and highest-altitude nations in Asia—showed COPD burdens above the regional median. Reviews of high-altitude populations have demonstrated that solid-fuel heating during cold seasons, combined with unfavorable meteorological conditions, leads to persistently elevated indoor PM₂.₅ levels, which are strongly associated with increased COPD risk (32, 33). Regarding occupational factors, a meta-analysis in Central Asia revealed a significant association between exposure to vapors, gases, dusts, and fumes and COPD risk, with a stronger effect observed in Kazakhstan (34, 35). Although Kazakhstan ranks only 25th in Asia by population, this study showed that its COPD burden is among the highest. During the heating season, PM₂.₅ concentrations in Almaty frequently reach extreme levels, with daily AQI values ranking among the world’s highest (36). Combined with emissions from mining and oil, and gas industries, urban air pollution has worsened (37). Given the difficulty of avoiding short-term high pollution events, emission reduction from biomass fuels, adoption of clean heating alternatives, industrial emission control, and enhanced public risk communication are essential.

In Southeast Asia, Indonesia, the world’s fourth most populous country, had 62.9% of men and 4.8% of women aged 15 years or older using tobacco (38). In addition to smoking, indoor air pollution from household biomass fuel use and occupational dust/fume exposures were also linked to increased COPD risk and should be prioritized in national-level interventions (39). In East Asia, North Korea has become a high-burden region due to severe carbon monoxide and aerosol pollution, highlighting the importance of energy structure and environmental governance (40). By contrast, Singapore achieved remarkable success in COPD prevention and control. From 1990 to 2021, its COPD burden declined significantly, benefiting from early diagnosis, tobacco-control legislation, comprehensive management strategies, and optimized healthcare resources (41, 42). These achievements not only highlight the effectiveness of systemic interventions but also provide transferable lessons for other countries.

Overall, the COPD burden in Asia shows striking heterogeneity. In South Asia, smoking and biomass fuel use dominate; in Central Asia, high altitude and occupational exposures are key drivers; in Southeast Asia, risks reflect both urban–rural disparities and environmental challenges linked to rapid urbanization; and in some regions, sustained declines have been observed due to early diagnosis, tobacco-control policies, and integrated disease management. Future prevention and control efforts should be tailored to local contexts, focusing on modifiable key risk factors to reduce regional burdens.

Analyses by sex and age demonstrated that COPD burden increased substantially with age, particularly after 45 years. Natural declines in lung function with aging, combined with chronic airway inflammation and parenchymal destruction, are the main contributors to increased burden among older adults (43, 44). Men bear a heavier burden due to historically higher smoking prevalence, whereas women are emerging as a potential high-risk group in countries where female smoking prevalence is rising (45, 46). These findings are consistent with a previous study on young COPD, which indicated that early-onset COPD already poses a public health threat and projected a continued increase in burden through 2050, suggesting a cumulative effect across the life course (47) Building upon this evidence, our study extends the analysis to middle-aged and older populations, confirming the significant increase in burden in the context of population aging and revealing divergent trends between men and women. Therefore, future prevention strategies should particularly target middle-aged and older men, as well as women, in settings with increasing smoking prevalence, to achieve more precise stratified interventions.

Joinpoint regression analysis showed that the COPD burden in Asia declined markedly between 2004 and 2007. This change was closely linked to the implementation of comprehensive interventions. Measures such as higher tobacco taxes, comprehensive smoke-free legislation, and improvements in air quality effectively reduced the COPD burden (48, 49). In addition, strengthening healthcare systems and optimizing chronic disease management promoted screening and early diagnosis, further reducing disease burden (50). Looking forward, continued intensification of tobacco control, optimization of healthcare services, and air quality improvements are essential to further mitigate the COPD burden in Asia.

In summary, this study demonstrates that COPD remains a substantial burden in Asia, with significant regional disparities driven by smoking, biomass fuel use, occupational exposures, and population aging. Clinically, early identification of high-risk populations and management of comorbidities should be prioritized, with special attention to rural and resource-limited populations and smokers. From a public health perspective, dual-track strategies are needed: strengthening tobacco control (taxation, smoke-free policies, cessation services) and systematically advancing air pollution control. Specifically, low-income countries should prioritize tobacco control and the adoption of clean energy and stoves; rapidly urbanizing countries should focus on PM₂.₅ control and tailored interventions for urban–rural differences; and high-SDI regions should continue optimizing early diagnosis and integrated chronic disease management. Although the relative contribution of occupational dust exposure may decline with industrial mechanization, population aging and persistent air pollution will remain major drivers of COPD burden, necessitating ongoing monitoring and dynamic policy adjustments.

This study has limitations. Data quality and availability were limited in some countries, which may affect the precision of estimates. Future work should rely on higher-quality epidemiological data and high spatiotemporal resolution exposure monitoring to support policy evaluation and effectiveness verification.

## Data Availability

The original contributions presented in the study are included in the article/Supplementary material, further inquiries can be directed to the corresponding author.

## Footnotes

^1^ http://ghdx.healthdata.org/gbd-results-tool

^2^ healthdata.org
