# Supplementary figures and images for: The burden and risk factors of chronic obstructive pulmonary disease in Asia and its countries from 1990 to 2021: a systematic analysis based on the 2021 global burden of disease study
Source: Front Med (Lausanne). 2025 Sep 19;12:1641719. doi: 10.3389/fmed.2025.1641719 (PMC12491052; doi:10.3389/fmed.2025.1641719)

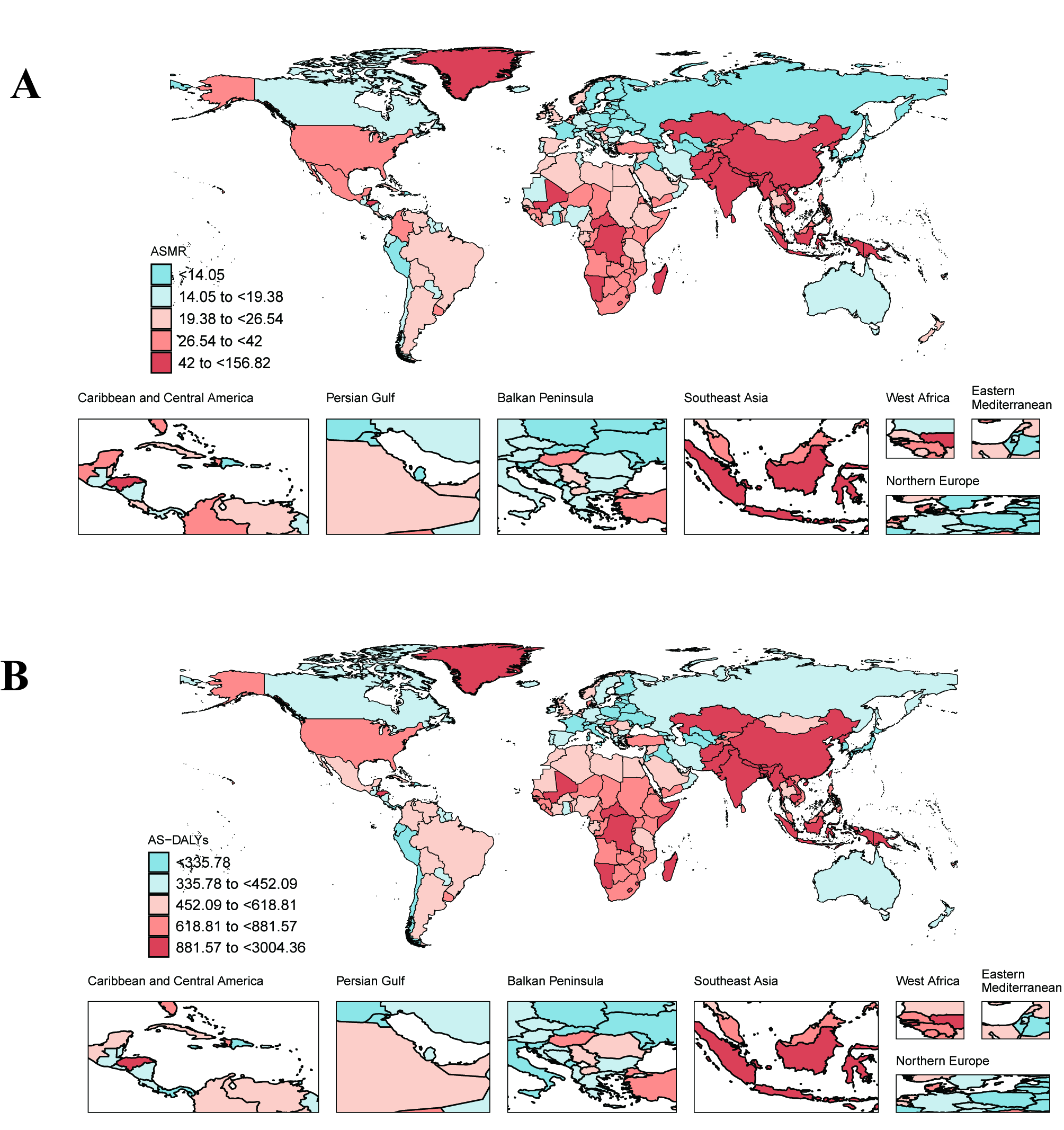

Supplement: Supplementary file 2 [file Image_1.tif]

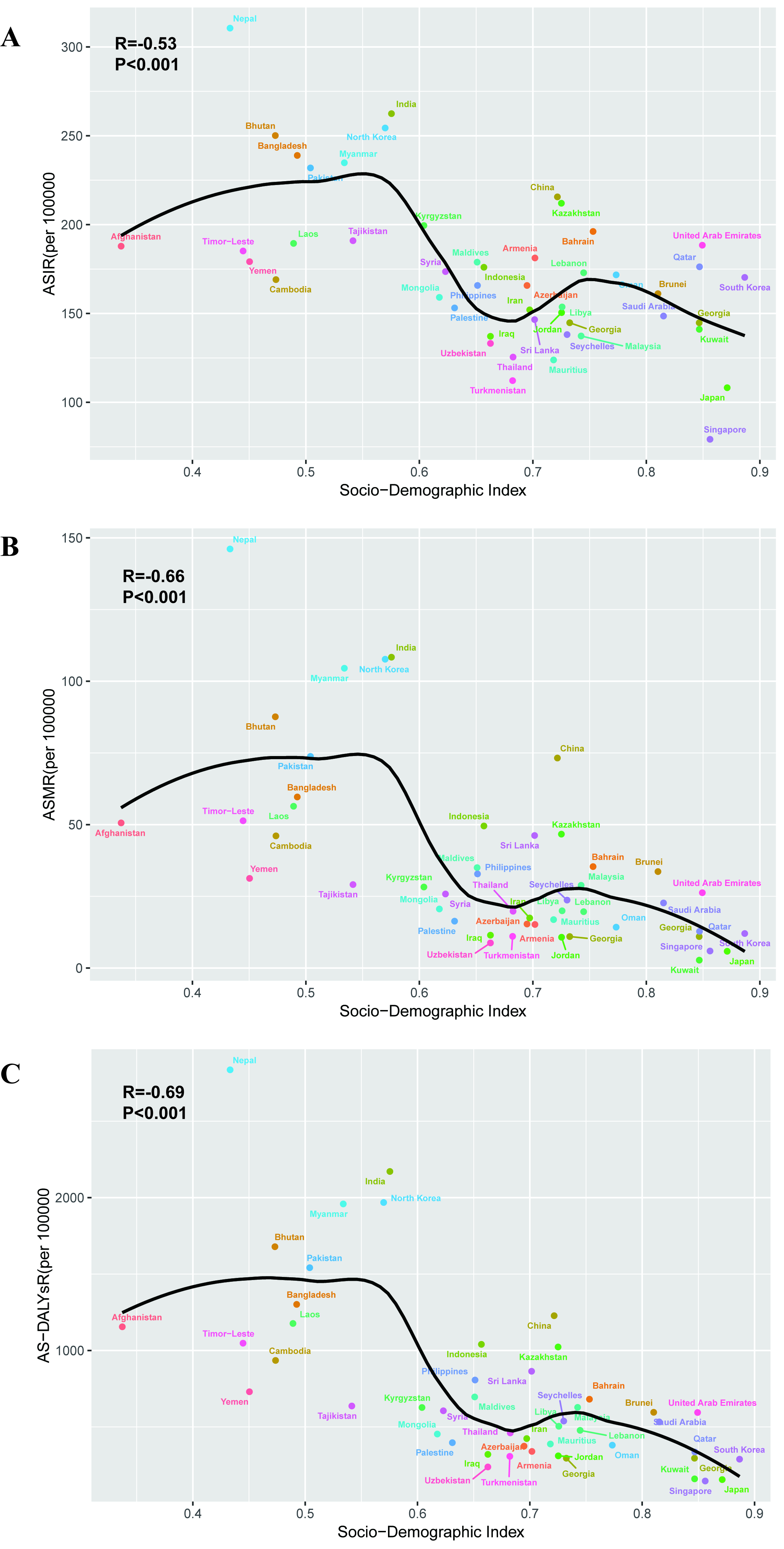

Supplement: Supplementary file 3 [file Image_2.tif]

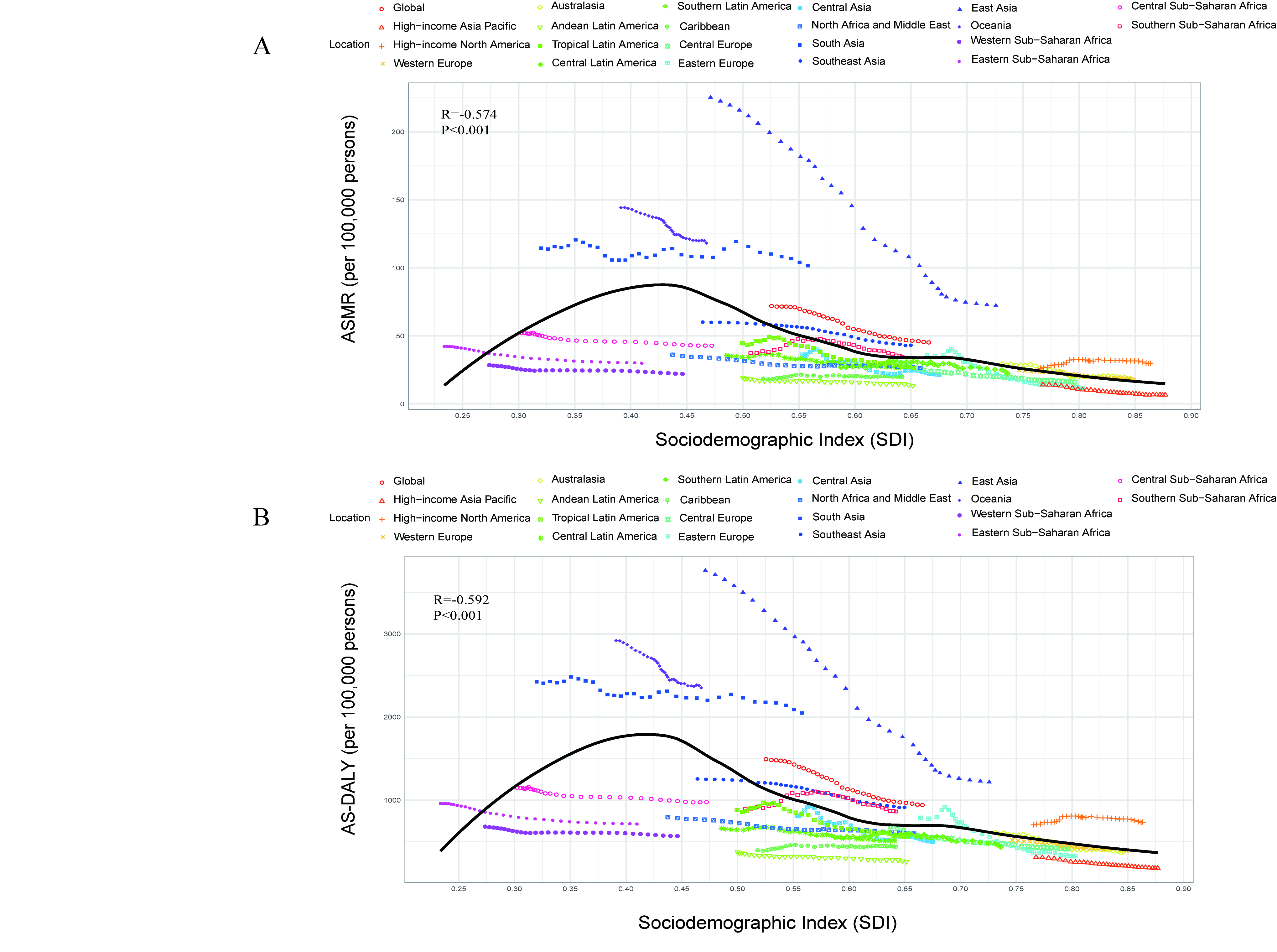

Supplement: Supplementary file 4 [file Image_3.tif]

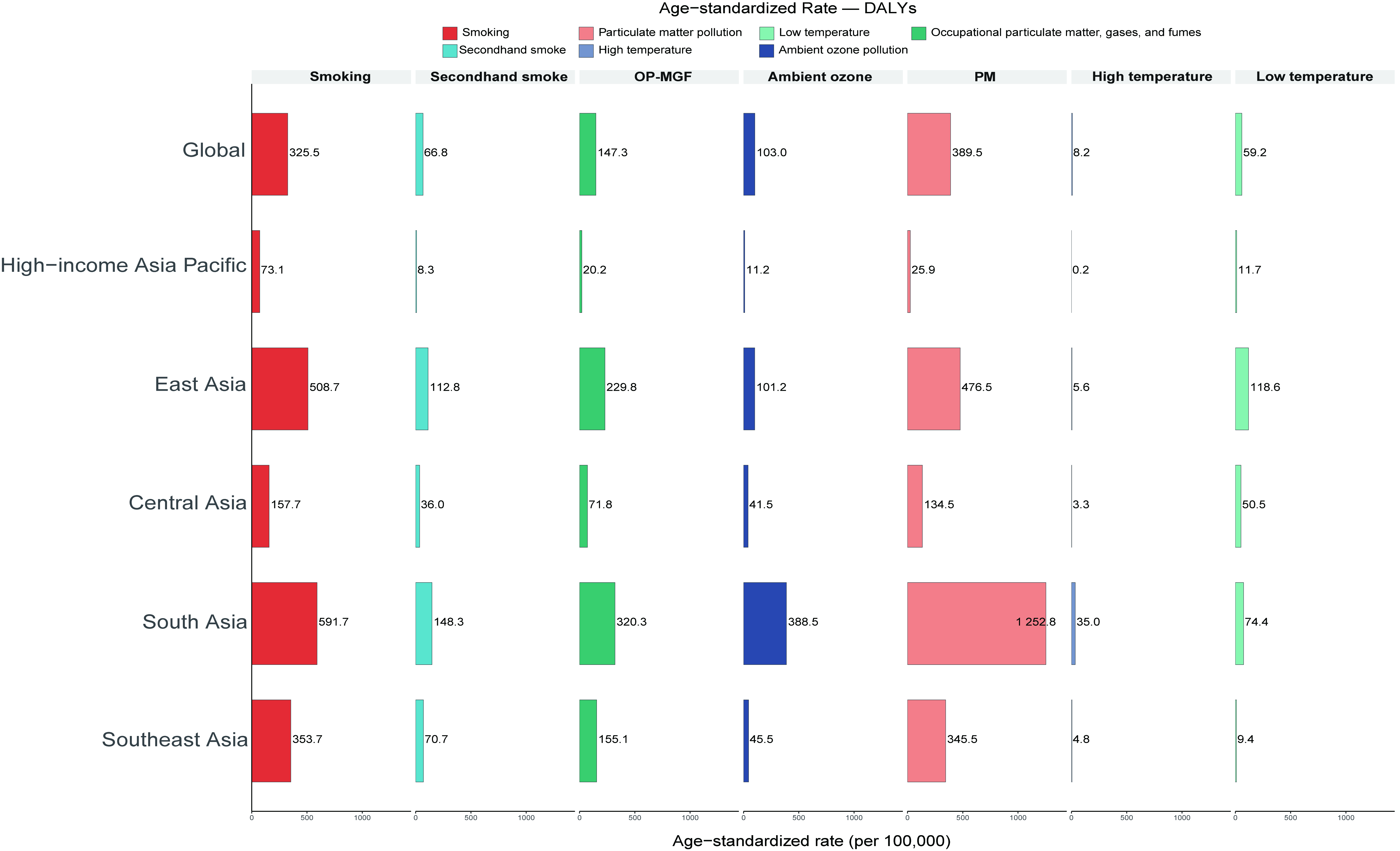

Supplement: Supplementary file 5 [file Image_4.tif]

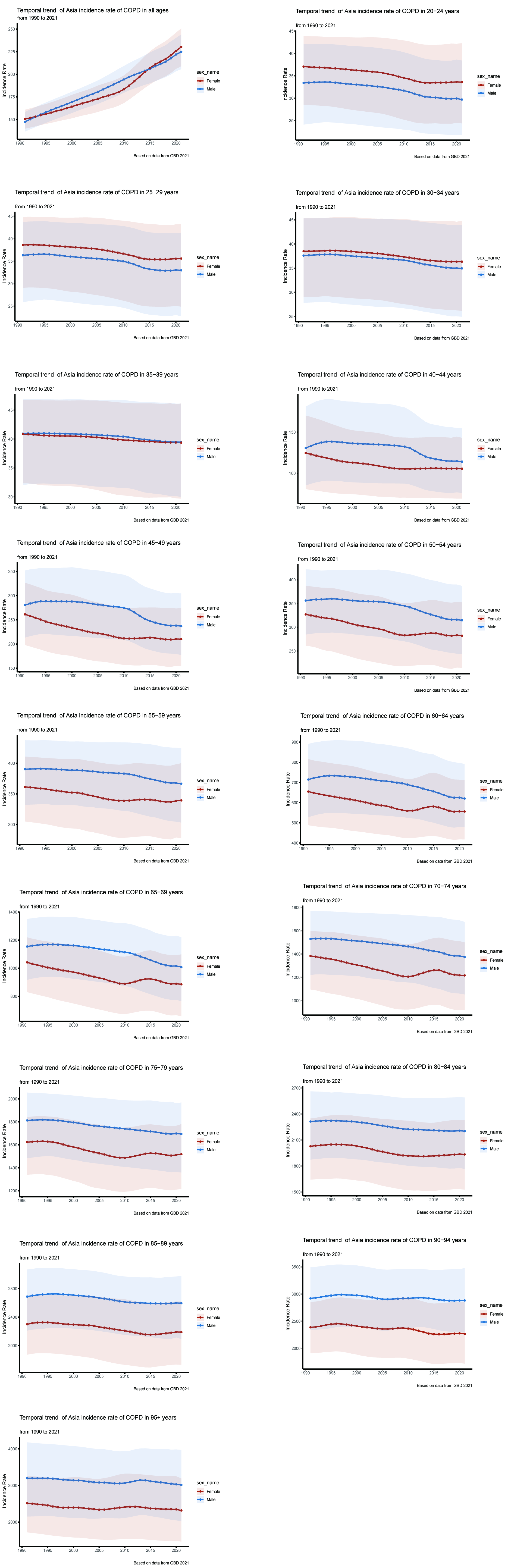

Supplement: Supplementary file 6 [file Image_5.tif]

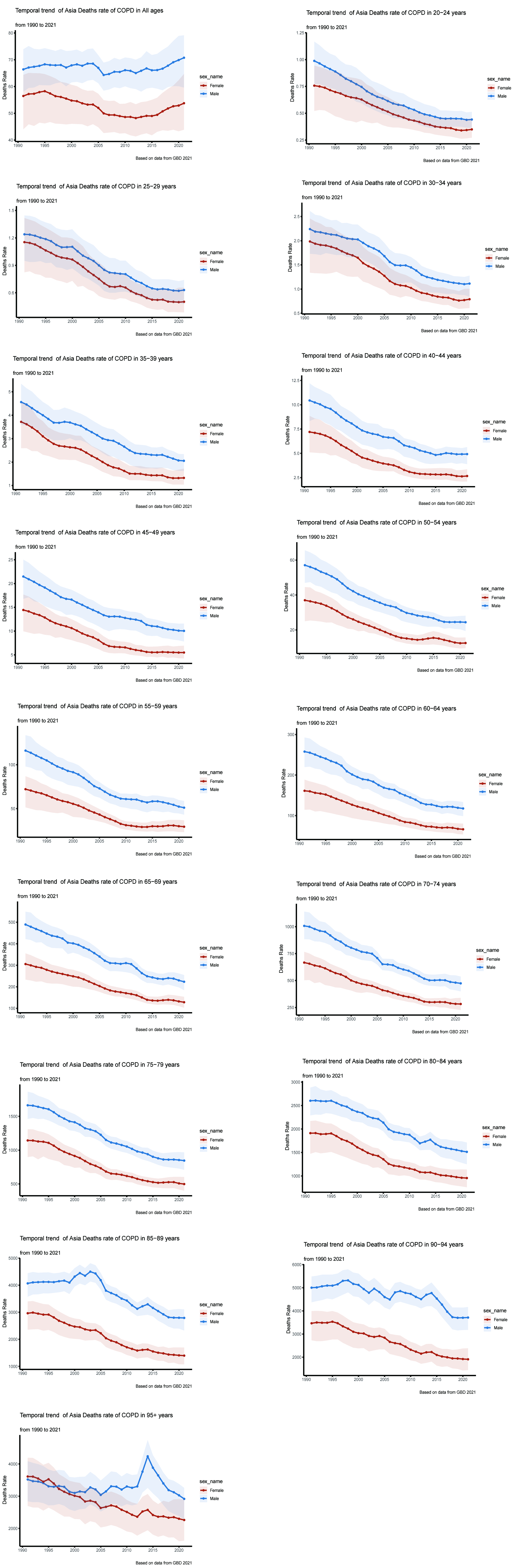

Supplement: Supplementary file 7 [file Image_6.tif]

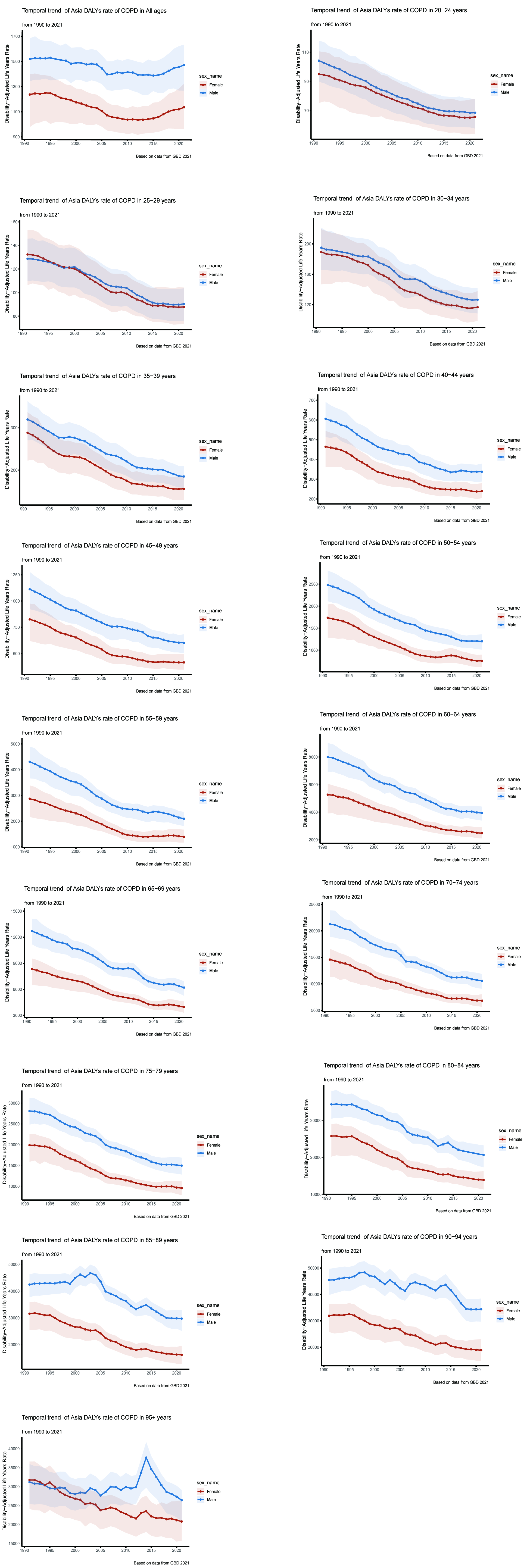

Supplement: Supplementary file 8 [file Image_7.tif]
